# Interleukin-18 and IL-18BP in inflammatory dermatological diseases

**DOI:** 10.3389/fimmu.2023.955369

**Published:** 2023-01-18

**Authors:** Xiaoyun Wang, Lian Wang, Xiang Wen, Lu Zhang, Xian Jiang, Gu He

**Affiliations:** ^1^Department of Dermatology, West China Hospital, Sichuan University, Chengdu, China; ^2^Laboratory of Dermatology, Clinical Institute of Inflammation and Immunology, Frontiers Science Center for Disease-Related Molecular Network and State Key Laboratory of Biotherapy, West China Hospital, Sichuan University, Chengdu, China

**Keywords:** IL-18 binding protein, IL-18, psoriasis, atopic dermatitis, rosacea, bullous pemphigoid, inflammatory dermatological disease

## Abstract

Interleukin (IL)-18, an interferon-γ inducer, belongs to the IL-1 family of pleiotropic pro-inflammatory factors, and IL-18 binding protein (IL-18BP) is a native antagonist of IL-18 *in vivo*, regulating its activity. Moreover, IL-18 exerts an influential function in host innate and adaptive immunity, and IL-18BP has elevated levels of interferon-γ in diverse cells, suggesting that IL-18BP is a negative feedback inhibitor of IL-18-mediated immunity. Similar to IL-1β, the IL-18 cytokine is produced as an indolent precursor that requires further processing into an active cytokine by caspase-1 and mediating downstream signaling pathways through MyD88. IL-18 has been implicated to play a role in psoriasis, atopic dermatitis, rosacea, and bullous pemphigoid in human inflammatory skin diseases. Currently, IL-18BP is less explored in treating inflammatory skin diseases, while IL-18BP is being tested in clinical trials for other diseases. Thereby, IL-18BP is a prospective therapeutic target.

## 1 Introduction

Interleukin (IL)-18, first described as an interferon (IFN)-γ inducer, is a member of the interleukin-1 (IL-1) cytokine family. IL-1 cytokines and their receptors are linked to inflammation, fever, and immunostimulation (1). There are 11 known members of the IL-1 family termed IL-1α, IL-1β, IL-1 receptor antagonist (Ra), IL-18, IL-33, IL-36α, IL-36β, IL-36γ, IL-36Ra, IL-37, and IL-38, of which IL-1α, IL-1β, IL-18, IL-33, IL-36α, IL-36β, and IL-36γ are pro-inflammatory and IL-1Ra, IL-36Ra, IL-37, and IL-38 are considered anti-inflammatory (2). The IL-1 and its related family member IL-18 lack signal peptides and are not readily secreted, except for IL-1Ra. IL-1 family proteins are synthesized as the inactive precursors in the cytoplasm, and the precursors contain consistent conserved sequence A-X-D, where A is likely to be an aliphatic amino acid, X represents any amino acid, and D stands for aspartic acid (2, 3). Furthermore, like IL-1β, the IL-18 precursor is processed by the IL-1β converting enzyme (ICE, caspase-1) to produce mature and active IL-18 (4). The IL-1 family members all have their corresponding receptors, and IL-1 and IL-18 receptors belong to the IL-1 receptor family (containing the Toll/IL-1 receptor (TIR) domain) (5). However, IL-18 activity can be neutralized, and the interaction with IL-18R is prevented by a natural inhibitor, IL-18 binding proteins (IL-18BP) (6).

Although discovered only 30 years ago, IL-18 has been reported as a versatile cytokine that causes a multitude of biological effects associated with infection, inflammation, and autoimmune processes, including inflammatory bowel diseases (7, 8), chronic liver disease (9), adult-onset Still’s disease (10), hemophagocytic syndrome (11), rheumatoid arthritis (12), idiopathic thrombocytopenic purpura (13), cancer (14, 15), and cardiac diseases (16). Furthermore, IL-18 and IL-18BP are also implicated in developing psoriasis, atopic dermatitis (AD), lupus erythematosus (LE), and other inflammatory skin diseases. In the past years, researchers reviewed the roles of IL-18 and IL-18BP on a diversity of skin diseases (17, 18). However, with new advances and discoveries, to our knowledge, the detailed roles of IL-18 and its binding protein signaling in skin diseases have not yet been thoroughly investigated. This literature review will address the most recent updates regarding IL-18 and IL-18BP in psoriasis, atopic dermatitis, rosacea, and bullous pemphigoid and discuss the underlying impacts of IL-18 and IL-18BP and inflammatory disease in the skin.

## 2 IL-18 and IL-18BP

### 2.1 IL-18

IL-18, first termed IFN-γ inducible factor (IGIF), has profound effects on natural killer and T-helper (Th) cell activation (19, 20). Furthermore, IL-18 is expressed constitutively in keratinocytes, macrophages, Langerhans cells (LC), dendritic cells (DC), as well as epithelial cells in the lack of an inflammatory stimulus (17, 21, 22). *IL-18* gene is located on chromosomes 11 and 9 in humans and mice, respectively (23). Besides, IL-18 protein is expressed at high concentrations in human keratinocyte lysates and is released as an unprocessed 24-kDa form (21). This gene encodes IL-18 protein and exists as pro-IL-18, mainly localized in the cytoplasm. Precursor human IL-18 (prohIL-18) has 193 amino acids with 24-kDa molecular weight converted into an active 18-kDa monomer of 157 amino acids by the cysteine proteinase caspase1-mediated cleavage of an N-terminal fragment (24). Precursor murine IL-18 (mIL-18) has 192 amino acids, and mature mIL-18 is a polyfunctional cytokine with 157 amino acids (25).

IL-18 is composed of 12 chains (S1-S12), forming three twisted 4-strand β-folded sheets, one short α-helix (H1) and one 3_10_-helix (H2). The three β-sheets fold over one another to form a β-trefoil fold. IL-18 shares sequence similarity (17%) with IL-1β of the IL-1 family, and both are structurally related as β-pleated sheet folded molecules (26). However, IL-18 and IL-1β show significant differences in the length and conformation of S3-S4, S4-S5, S7-S8, and S11-S12 segments (27). The essential residues of IL-18 and IL-1β make up sites I, II, III, and sites A, and B, respectively. Site I comprises Arg13, Asp17, Met33, Asp35, and Asp132 residues and is positioned on one side of the β-triplet folding central barrel, while site II consists of six residues (Lys4, Leu5, Lys8, Arg58, Met60, and Arg104) and is situated at the upper part of the β-barrel. Comparing the primary and tertiary structures of IL-18 and IL-1β indicates that sites I and II of IL-18 are in equivalent positions on the sequence and tertiary structure as sites A and B of IL-1β. Lys79, Lys84, and Asp98 are assembled at the bottom of the barrel, on the opposite side of site II, which is site III. Sites I and II are associated with IL-18Rα binding, whereas site III is involved in cellular responses (27, 28).

Pro-IL-18 processing, maturation, and activation of IL-18 are primarily mediated through canonical caspase-1 or IL-1β-converting enzyme induced by inflammasomes. Formation of inflammasomes, including members of leucine-rich-repeat-(LRR)-NOD-like receptors (NLR) family and the absence in melanoma 2 (AIM2) in recognition of microbial or danger signals, leads to the procaspase-1 cleavage into active caspase-1 enzyme, the intracellular cysteine protease, further cleaving pro-IL-18 into their mature molecule forms (32, 40). Moreover, NLRP6 (PYD domains-containing protein 6) can recruit caspase-1 and atypical caspase-11. Upon recruitment, caspase-11 is processed and stimulates caspase-1-dependent IL-18 secretion (39). NLR family CARD domain containing-4 (NLRC4) also can mediate IL-18 processing *via* caspase-1 in immune cells (46). In addition, other alternative pathways regulated by inflammatory cells can also cause the synthesis and secretion of active IL-18. For example, fas-ligand signaling can trigger caspase-8 in dendritic cells and macrophages, which leads to the processing and release of mature IL-18 (33). Furthermore, IL-18 binding to IL-3 can lead to the release of mast cell-induced chymase, and in turn, pro-IL-18 can be activated by chymase (34). And Granzyme B (GrB), a family member of serine proteases, converts proIL-18 into mature IL-18 in non-hematopoietic cells such as keratinocytes (35, 36). IL-18 activity is regulated at the transcriptional and promoter level (37), by post-translational modification (caspase-1 cleavage), and by binding of the endogenous inhibitor IL-18BP (29).

### 2.2 IL-18 receptor

After maturation and activation, IL-18 participates in several biological functions through the IL-18 receptor chains (IL-18R). IL-18 combines with the IL-18R which comprises two subunits, one for the IL-18 receptor conjugated α chain (IL-18Rα) and the other for the non-conjugated signaling chain (IL-18Rβ). Furthermore, IL-18R is a heterodimeric complex that is a member of the IL-1 receptor family (30). IL-18Rα is expressed by most cells, while only a few cells, like T cells, dendritic cells, and mast cells, express the IL-18Rβ chain (31).

**Figure 1:**
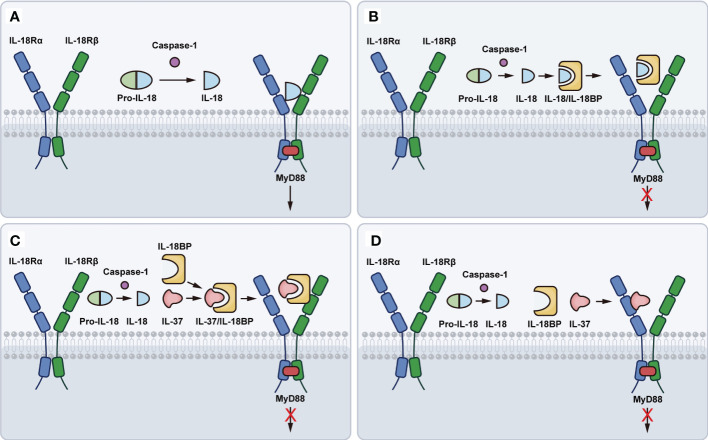
Schematic representation of IL-18 binding to IL-18R. **(A)** The pro-IL-18 is processed by the caspase-1 to produce mature and active IL-18. Mature IL-18 links to the low-affinity IL-18Rα to form an inactivated IL-18/IL-18Rα binary complex, which recruits the IL-18Rβ chain to develop a high-affinity complex that launches IL-18-dependent signaling pathway. **(B)** IL-18BP binds to IL-18 and further forms a blunting complex with IL-18Rα, preventing IL-18Rβ from activating cells. **(C)** IL-37 binding to IL-18BP, forms a complex with IL-18Rβ, thereby enhancing the inhibition of IL-18 activity. **(D)** IL-37 binds to IL-18Rα, causing the loss of recruitment with IL-18Rβ.

The ligand-binding IL-18Rα has the same chromosomal location as IL-1R types I and II, of which ectodomain has three Ig-like domains (D1, D2, and D3) in a curved shape. The D1 and D2 domains are tightly aligned, forming the D1D2 module, connecting to the D3 domain *via* a Linker. Each of the three domains possesses a bilayer sandwich structure consisting of 6-9 β-strands and at least one intradomain disulfide bond. IL-18Rα has a similar structure to IL-1RI. The three domains of IL-18Rα form a grasping hand that wraps IL-18 inside (41). The IL-18Rβ (also known as IL-18 receptor accessory protein-like (AcPL) molecule) is associated with IL-1R accessory protein (42) and is required for signaling (43). IL-18Rβ also contains three Ig-like domains, similar to those of IL-18Rα, except for the disulfide bonds within the domain and the spatial arrangement of the three domains in the triplet compound. IL-18Rβ D2 and D3 are closely coupled and directly adjacent to IL-18/IL-18Rα, whereas D1 is not relevant for the formation of the ternary compound. Further, the IL-18 and IL-18Rα complex concave surfaces, where the IL-18 site III and D2 of IL-18Rα are located, match the convex surfaces of IL-18Rβ-D2 (44). In a word, IL-18 activates the downstream signaling process by linking to the low-affinity IL-18Rα to form a signaling complex, producing an inactivated IL-18/IL-18Rα binary complex, which recruits the IL-18Rβ chain to develop a high-affinity complex that launches IL-18-dependent signaling pathway (45) (**Figure 1A**). In the presence of IL-18Rβ deletion, IL-18/IL-18Rα complex would not be a pro-inflammatory signal.

### 2.3 IL-18 binding protein

IL-18BP, with 40-kDa weight, belongs to the Ig superfamily and is a decoy receptor of IL-18. It resembles the extracellular structural domain of the immunoglobulin (Ig)-like receptor but without a trans-membrane and cytosolic domain (4, 38). Previous studies have found that human and murine IL-18BP (huIL-18BP and muIL-18BP) are homologous to molluscum contagiosum virus (MCV) and poxviruses, including smallpox virus (47). Soluble IL-18BP is induced by IFN-γ and is constitutively expressed and secreted by the human spleen and leukocytes (4, 48). Efficient production of IL-18BP may counteract excess IL-18 in epithelial cells and antagonizes the pathological inflammation at the biological barrier (49). Four isoforms of human IL-18BP (IL-18BP a, b, c, and d) have been identified from the alternative mRNA splicing of the human gene, which is located on chromosome 11q13 and has no exon coding for a transmembrane domain. IL-18BP a and c both have intact Ig domains that neutralize mature IL-18, whereas the non-binding isoforms b and d lack a complete Ig domain at C-terminus, and their biological function is currently unknown (50). Usually, IL-18BPa represents IL-18BP due to its high affinity to IL-18 (51). There are two isoforms in mice, IL-18BPc and IL-18BPd, which possess the identical Ig domain and neutralize more than 95% of murine IL-18 (50).

Based on the resolved IL-18BP of ectromelia virus (ectv), ectvIL-18BP was concluded that it comprised of two 4-stranded β-sheets with a short c’ strand, a helix (H1), and elongated flexible loops between the β-sheets (52). IL-18BP can balance IL-18-driven activation by binding mature IL-18, thereby blocking its interaction with the activated receptor IL-18R, and this affinity is higher than IL-18Rα (4). IL-18BP has been demonstrated to bind to IL-18 using a single Ig fold and interact with IL-18 in a manner closely resembling the D3 domain of IL-18Rα signaling, thus directly preventing the binding site II between IL-18 and IL-18Rα (41, 52–54). Furthermore, IL-18BP binds to IL-18 and further forms a blunting complex with IL-18Rα, preventing IL-18Rβ from activating cells (55, 56) (**Figure 1B**).

IL-18BP can also bind to IL-37, another anti-inflammatory cytokine of the IL-1 family. IL-37, binding to IL-18BP, forms a complex with IL-18Rβ, depriving it of the β-chain that forms a complex with IL-18Rα as a functional receptor, thereby enhancing the inhibition of IL-18 activity (57, 58) (**Figure 1C**). Besides, a recent study indicates that IL-37 can bind to IL-18Rα and act as a negative regulator, causing the loss of recruitment with IL-18Rβ (59) (**Figure 1D**). Under physiological conditions, plasma IL-18BP concentration is much higher than IL-18, preventing IL-18 from binding to IL-18Rα and IL-18Rβ. Therefore, the concentration of plasma IL-37 depends on IL-18BP. Indeed, IL-18BP has been reported to be a potential treatment for several diseases. IL-18BP is an immunoregulatory binding protein and natural product with attractive therapeutic effects for diseases that are mediated, in part, by IL-12, IFN-γ, or IL-18 itself (38). Furthermore, IL-18BP is protective against LPS-induced acute lung injury, which may be related to its regulation of NF-κB and nuclear factor erythroid 2-related factor 2 (Nrf2) activity (60). In addition, downregulation of IL-18BP leads to elevated vascular cell adhesion molecule 1 (VCAM-1) expression, monocyte/macrophage adhesion, and accelerated atherosclerotic plaque formation in diabetic mice (60).

## 3 Signaling and function of IL-18 and IL-18BP

**Figure 2:**
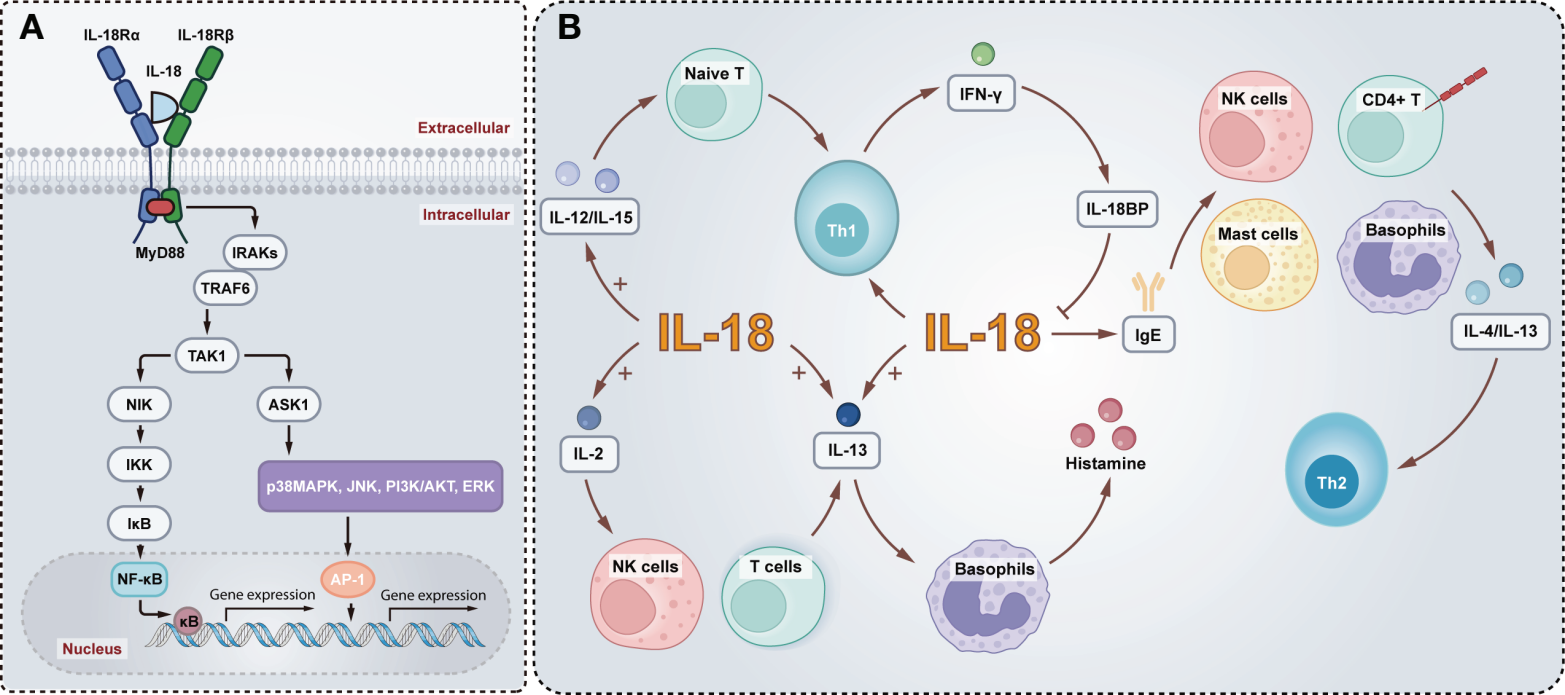
Schematic model of the IL-18 signaling pathways and biological functions. **(A)** Active IL-18 is secreted extracellular, and activation of IL-18R recruits MyD88 into the TIR domain and anchors IRAKs, which bind to TRAF6 associating with TAK1. TAK1 in turn phosphorylates NIK. This is followed by the activation of IKK, phosphorylation of IκB, and finally nuclear translocation of NF-κB, which regulates the transcription of inflammatory genes. In addition, the TRAF6 activates ASK1 through TAK1, and contributes to activate p38MAPK, JNK, PI3K/AKT and ERK, leading to the activation of AP-1. **(B)** IL-18 enhances Th1 production of IFN-γ which in turn induces IL-18BP inhibits IL-18. Mature IL-18 with IL-12 or IL-15 induces differentiation of naive T cells into Th1 cells. Alternatively, IL-18 alone elevates IgE and actives mast cells, basophils, NK cells, and CD4^+^ T cells to produce IL-13 and IL-4, driving a Th2 response. Furthermore, IL-18 and IL-2 promote IL-13 production in NK cells and T cells and, in conjunction with IL-13, induce histamine release from basophils.

The signaling transduction pathways of IL-18 are similar to IL-1β. Analogous to the IL-1β receptor IL-1R, IL-18R includes a Toll/IL-1 receptor (TIR) domain in the cytosolic region, so the signal into the cell is first mediated by a molecule known to be an adaptor of TLRs and IL-1R, myeloid differentiation 88 (MyD88), which docks with the IL-18R complex and leads to the recruitment and phosphorylation of IL-1 receptor-associated kinases (IRAKs). Both IRAK1 and IRAK4 are implicated in IL-18 signaling in Th1 and NK cells (61–63). The IRAKs bind to another adaptor molecule, TNF receptor-associated factor 6 (TRAF6), which associates with a kinase called TAK1 (transforming growth factor-β-activated kinase 1), the kinase that in turn phosphorylates nuclear factor kappa B (NF-κB)-induced kinase (NIK). Moreover, NIK activates the I kappa B (IκB) kinase (IKK), which phosphorylates IκB, causing IκB ubiquitination and rapid degradation. Transcription factor NF-κB is then free and migrates into the nucleus, where it combines with specific regulatory sequences (κB sites) in the promoter regions of multiple inflammatory genes (*e.g.*, IFN-γ, IL-8, IL-1β, TNF-α) (64–68). Furthermore, the phosphorylation of TRAF6 also associates with apoptosis signal-regulating kinase 1 (ASK1) *via* TAK1, and interfaces with ASK1 causing the activation of mitogen-activated protein kinases (MAPK)-related signaling downstream, p38 mitogen-activated protein kinase (p38MAPK), jun kinase (JNK), phosphoinositide 3-kinase (PI3K)/AKT, extracellular regulated protein kinases (ERK), resulting in the activation of AP-1 transcription factor (65, 69) (**Figure 2A**).

Besides, IL-18 can enhance Th1 responses and is essential for IFN-γ production (70). Conversely, the IFN-γ-induced soluble IL-18BP inhibits the biological activity of IL-18, thereby reducing IFN-γ production and thus suppressing the Th1 immune responses. It is readily speculated that a dysregulation of the IL-18/IL-18BP balance may contribute to chronic inflammation driven by the T-helper type I cytokine. Indeed, IL-18 has the opposite impacts on Th1 and Th2 activation balance (71). Mature IL-18 with IL-12 or IL-15 induces differentiation of naive T cells into Th1 cells. However, administration of IL-18 alone mainly results in elevating IgE expression levels and enhanced IL-4 and IL-13 production by mast cells, basophils, NK cells, and CD4^+^ T cells, driving a Th2 response (72–74). Furthermore, IL-18 and IL-2 promote IL-13 production in NK cells and T cells. And IL-18 induces histamine release from basophils in concert with IL-13 (75) (**Figure 2B**). IL-18 enhances Fas Ligand (FasL) on natural killer (NK) cells as well, thereby facilitating FasL-mediated NK cytotoxicity (29).

Furthermore, IL-18 can promote allergic inflammation through mast cells and basophils to enhance the production of critical factors that drive atopic inflammation (76). This evidence suggests the pleiotropic function of this cytokine in promoting immune responses and inflammation, with IL-18 acting as a key bridge between innate and adaptive immunity. IL-18 has been found to have two biological functions, first as a co-stimulator of Th1 cytokine production, together with IL-2, IL-12, microbial agents, or mitogens to promote the production of IFN-γ. In addition, IL-18 directly induces tumor necrosis factor (TNF)-α, IL-1β, CXC, and other chemokines, Fas ligand, and vascular adhesion molecules, and NF-κB nuclear translocation, initiating cytokine cascade responses accompanied by the expression of several pro-inflammatory markers, possibly as a contributor to inflammation (6, 56, 77).

### 3.1 IL-18 and IL-18BP in keratinocytes

Keratinocytes (KC) represent makeup 95% of the epidermis and are considered to play a critical role in skin inflammation and immune response. In human KC, pro-IL-18 is constitutively expressed (22), and compared to monocytes, PBMC, or leukocytes, keratinocytes produce large amounts of pro-IL-18 (78). It has been found that in human keratinocyte cell line HaCaT, ultraviolet B (UVB) irradiation time- and dose-dependently promotes the production of IL-18, which is selectively mediated through reactive oxygen intermediates (ROI) production and activator protein-1 (AP-1) activation (79). IL-18R is also present in keratinocytes, signifying that IL-18, released from keratinocytes, acts in an autocrine or paracrine manner on surrounding keratinocytes (80). Besides, INF-γ significantly upregulates IL-18BPa mRNA expression in HaCaT cells, while IL-18 mRNA expression levels were not affected (81).

In addition to their barrier function, epidermal keratinocytes can sense harmful pathogens through pattern recognition receptors (PRRs). Besides Toll-like receptors (TLRs), epidermal keratinocytes also have nucleotide-binding oligomerization domain (NOD)-like receptors (NLRs), which is an intracellular PRR (82). Inflammasomes are large, complex proteins consisting of NLR, the adapter protein- ASC containing a carboxy-terminal caspase-recruitment domain of the apoptosis-associated speck-like protein, and caspase-1, an inducer of IL-1β and IL-18 that promotes inflammation and inflammatory cell death. NLR interacts directly with caspase-1 *via* caspase activation and recruitment domain (CARD) or by the ASC linking NLR to caspase-1 (83). There are five PRRs (NLRP1, NLRP3, NLRC4, pyrin, and AIM2) that form various inflammasomes. In addition, other members of the NLR family and the PYHIN family, including NLRP6, NLRP7, NLRP12, and IFI16 (γ-interferon-inducible protein 16), can also be found form different inflammasomes (84). Upon detecting danger signals, inflammasomes initiate the immune response by activating caspase-1 to cleave precursors of IL-1β and IL-18, releasing mature IL-1β and IL-18. Dai et al. found that cytoplasmic double-stranded RNA (dsRNA) is significant in mediating the activation of NLRP3 inflammasomes, causing the release of mature IL-1β and IL-18 and that cytosolic dsRNA-activated protein kinase (PKR) is a potent factor in human primary keratinocytes through dsRNA-mediated activation of NLRP3 inflammasomes (82). Besides, Papale et al. revealed that NLRP12 expression was inversely related to IL-18 production in KC (85). Zhang et al. demonstrated that activation of AIM2 by PKR upregulates the production of mature IL-1β and IL-18 in human keratinocytes (86). Fenini et al. found that NLRP1 inflammasomes have an essential function in UVB perception and subsequent secretion of IL-1β and IL-18 from keratinocytes (87). Moreover, Galbiati et al. speculated that contact allergens could induce oxidative stress, which activates inflammasome and releases high mobility group protein B1 (HMGB1), further activating TLR4 which leads to the neo-synthesis of IL-18 in human KC (88). The IFN-γ-induced chemokines CXCL9 (chemokine C-X-C motif ligand 9), CXCL10, and CXCL11 recruit Th1 cells by binding to CXCR3 (chemokine C-X-C motif receptor 3) on the cell surface (89) and significant infiltration of Th1 cells are seen in inflammatory skin diseases. In keratinocytes, IL-18 enhances the mRNA expression of CXCL9, CXCL10, and CXCL11 induced by IFN-γ. Further, it is suggested that IFN-γ activates STAT1 *via* JAK1/JAK2 and/or p38MAPK, leading to CXCL9, CXCL10, and CXCL 11 production. In addition, IFN-γ can also affect the activation of IRF-1 (interferon regulatory factor-1), which is only responsible for CXCL11 through p38MAPK, while IL-18 enhances this activation through PI3K/AKT and MEK/ERK. IL-18 induces NF-κB activity through MEK/ERK and PI3K/AKT pathways, thus enhancing IFN-γ-induced CXCL9 secretion (75).

In human dermal fibroblasts (HDF), IL-18 regulates transcription factor Ets-1 activation *via* the ERK pathway, thereby reducing collagen expression and inhibiting TGF-β-induced collagen production. Therefore, IL-18 exerts antifibrotic activities in dermal fibroblasts (90). The IL-18 expression level is reduced, indicating that IL-18 is closely associated with hypertrophic scar formation in hypertrophic scar fibroblasts and hypertrophic scar tissue. Recent studies have revealed that IL-18 facilitates apoptosis in hypertrophic scar fibroblasts by enhancing the production of Fas ligand (FasL), and rhIL-18 upregulates Caspase-3, Caspase-8, Caspase-9, and FADD (FAS-associated death domain) expression in a dose-dependent manner (91). FasL is a ligand for Fas that binds specifically to Fas, and FADD recognizes the death domain (DD), which in turn forms a death-inducing signal complex (DISC) with caspase-8, further activating caspase-3 and delivering it to the cytoplasm, thereby launching the caspase cascade and apoptosis (92). Consequently, rhIL-18 can inhibit cell proliferation in hyperplastic scars by increasing FasL expression to initiate apoptosis, and it is a promising target for the therapy of hyperplastic scars.

### 3.2 IL-18 and IL-18BP in immune cells

The keratinocytes are exposed to external irritation and release of IL-18, which acts on surrounding keratinocytes and affects immune cells (DC, LC, macrophages, among others) in the skin, thus causing an inflammatory response. Langerhans cells, a population of antigen-presenting and myeloid-derived immature dendritic cells, reside in the epidermis and are the first to encounter skin pathogens, and LC are activated into mature DC in response to various stimuli. IL-18 induces LC migration and DC accumulation, with mature LC migrating to skin-draining lymph nodes where they deliver antigens to CD4^+^ T cells and regulate the adaptive immune response. In addition, IL-18 acts synergistically with IL-12 to stimulate Th1 production of INF-γ. IL-18 does not induce Th1 differentiation but upregulates IL-12Rβ expression, thereby enhancing IL-12-mediated Th1 development (93, 94).

Neutrophils are participants in innate immunity and are responsible for bacterial killing when pathogens invade, influencing inflammatory and immune responses by producing large numbers of cytokines. In parallel to phagocytic and microbicidal features, neutrophils are essential in initiating and/or amplifying inflammatory and immune responses (95). Unstimulated neutrophils secrete IL-18BP and IL-18, a neutrophils activator, inducing the expression and release of multiple inflammatory cytokines in neutrophils (96, 97). Fortin et al. revealed that IL-18 induced the production of IL-8, CCL3/Mip-1α, CCL4/Mip-1β, and CCL20/Mip-3α from human neutrophils and affected the expression of IL-8, Mip-1α, and Mip-1β through the p38MAPK, ERK, PI3K/AKT, and IKK/NF-κB pathways (96).

In human peripheral blood mononuclear cells (PBMC), mature IL-18 induces IL-8 production. Furthermore, IL-18BP inhibits IL-12-induced IFN-γ generation in PBMC. IL-18 stimulation results in IL-8 synthesis increased in macrophages. IL-18 induces IFN-γ synthesis by T-cells and natural killer cells, whereas IL-12, mitogens, or microbial agents, are necessary for IL-18 to induce the production of IFN-γ (72).

## 4 IL-18 and IL-18BP in inflammatory dermatological diseases

To date, many reports revealed the dysregulation of IL-18 in inflammatory skin diseases, and IL-18 has been speculated to be a possible marker for diverse disorders. Here, the topic mainly focuses on psoriasis, atopic dermatitis, bullous pemphigoid, and rosacea; other disorders, including cutaneous lupus erythematosus, dermatomyositis, and allergic contact dermatitis, are also reviewed. IL-18 inhibitors and IL-18BP drugs in clinical trials and preclinical studies for the treatment of inflammatory skin diseases or related diseases were summarized in **Table 1**.

**Table 1:** Drugs targeting IL-18 and IL-18BP for inflammatory dermatological diseases and related diseases.

Drug	Target	mechanism	Status	Drug category	Indication
Tadekinig alfa (AB2 Bio SA)/r-hIL-18BP/recombinant Interleukin-18 Binding Protein	IL-18	IL-18 inhibitor	Phase III	recombinant human IL-18 binding protein	Macrophage Activation Syndrome/Immune System Diseases/Adult-Onset Still’s Disease (98, 99)
CMK-389	IL-18	IL-18 inhibitor	Phase II	Biological products	Atopic Dermatitis/Pulmonary Sarcoidosis
Camoteskimab/AEVI-007/CERC-007/MEDI-2338/	IL-18	IL-18 inhibitor	Phase I	Monoclonal antibody	Adult-Onset Still’s Disease/Autoimmune Diseases/
ST-067	IL-18	IL-18 agonist	Phase II	Biological products	Melanoma/Carcinoma/Non-Small Cell Lung Cancer
APB-R3/long acting IL-18 binding protein (Aprilbio)	IL-18	IL-18 inhibitor	Drug discovery	Fusion protein	Adult-Onset Still’s Disease/Rheumatoid Arthritis/Inflammatory Bowel Diseases
Tadekinig alfa (Yeda Pharma)	IL-18	IL-18 inhibitor	Phase II (terminated)	recombinant human IL-18 binding protein	Psoriasis/Rheumatoid Arthritis/Crohn Disease/Autoimmune Diseases
AMP18P1RA/IL-18bp-Fc-IL-1ra	IL-18, IL-1R1	IL-18 Inhibitor, IL-1R1 Antagonist	Preclinical (progression-free)	Fusion protein	Psoriasis/Myocardial Ischemia/Neoplasm Metastasis

### 4.1 Psoriasis

**Figure 3:**
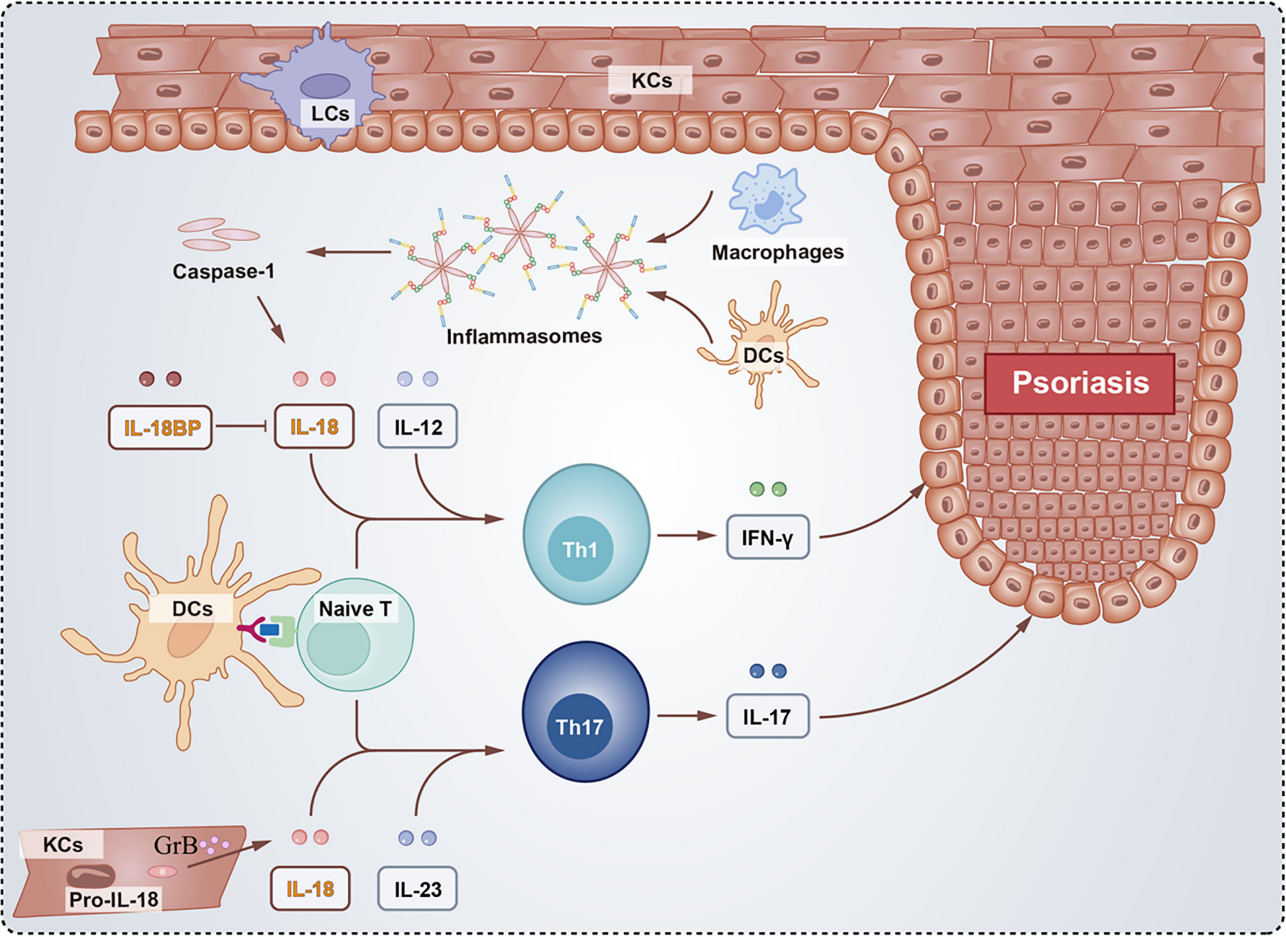
Schematic of the role of IL-18 and IL-18BP in psoriasis. The activated inflammasomes directly recruit and cleave procaspase-1 into caspase-1, whose proteolysis activate the pro-inflammatory cytokine IL-18. Besides, proIL-18 were cleaved by GrB in KCs. Inflammatory cytokines including IL-12 and IL-23 produced by Naïve T together with IL-18 facilitate the differentiation of Th1 and Th17 cells, respectively. The Th1 cells secrete IFN-γ and Th17 cells secrete IL-17 both contributing to psoriasis. However, IL-18BP binds to IL-18 blocking the development of psoriasis.

Psoriasis is a common cutaneous inflammatory disease with different variants, including plaque psoriasis, pustular psoriasis, guttate psoriasis, and erythrodermic psoriasis (100). The pathogenesis of genetics and immunity in psoriasis is widely accepted (101). Triggers from exogenous or endogenous environments in genetically susceptible individuals activate the immune system and activate cell networks, including keratinocytes, plasmacytoid dendritic cells, macrophages, and T cells, primarily the Th1 and Th17 pathways (102, 103) (**Figure 3**).

Many reports have focused on the significance of IL-18 in psoriasis. Patients with psoriasis had significantly higher levels of IL-18 in the lesions and in the serum compared to healthy controls (104). In previous studies, serum or plasma IL-18 concentration has been correlated with the severity of psoriasis and the Psoriasis Area and Severity Index (PASI), and therefore, IL-18 may be considered a possible biomarker of psoriasis (105–107). Recently, Deepti Verma et al. revealed that patients with psoriasis (without treatment) exhibited high plasma IL-1β and IL-18 levels, while normal plasma levels of IL-1β and IL-18 in psoriasis patients treated with anti-TNF agents (108). Moreover, Niu et al. explored the possible pathological mechanisms of IL-18 in psoriasis and reported that IL-18 might exacerbate pronounced inflammation and impact pathological features to cause micro-abscesses and scale formation through upregulating pro-inflammatory cytokines and reducing protective cytokines in a mouse model of imiquimod-induced psoriasis (109). Furthermore, a recent study found that recombinant mouse (rm) IL-18 synergized with rmIL-23 to induce prominent inflammation, upregulate the levels of IFN-γ and CXCL9, and enhance psoriasis-like epidermal hyperplasia, indicating that IL-18 might cooperate with IL-23 to induce a Th1 immune reaction, thereby aggravating psoriatic inflammation (110). Besides, IL-18 has been shown to facilitate the production and maintenance of Th17 cells, and Zhang et al. found that IL-18-neutralizing antibody could block the Th17 immune response in the psoriasis-like mouse model (46, 111). These pieces of evidence indicate that IL-18-mediated T cell response may hold an implicated role in psoriasis, and the inhibition of IL-18 can be a potential therapy for psoriasis.

### 4.2 Atopic dermatitis

Atopic eczema or atopic dermatitis (AD) is the most common inflammatory skin disorder. The primary pathogenesis of AD includes the skin barrier defect and immune dysregulation, especially innate and Th2 immune response (112). IL‐18 is a marker for innate immune activation, and many reports have indicated elevated serum levels of IL-18 in AD patients (113–115). Restriction fragment length polymorphism suggested that GG genotype was correlated significantly with elevated serum IL-18 levels, and the G allele of the IL-18 gene (rs 187238) was a risk factor for AD (116). GG genotype of IL-18 in AD patients was related to elevated levels of IgE and pruritus. GG genotype and G-allele in the -137 position of IL-18 increased the risk of AD in the Polish population (117, 118). Besides, Inoue Y et al. revealed consistently upregulated IL-18 levels in the horny layer of skin lesions in AD patients than in healthy controls, and IL-18 levels in the horny layer of patients colonized with *Staphylococcus aureus* (*S. aureus*) were significantly higher compared with those who were not (119). Moreover, McAleer MA et al. measured multiple cytokines and chemokines in the stratum corneum and plasma from AD and healthy children (120). As far as these cytokines are concerned, IL‐18 was the most significantly elevated biomarker in the AD skin compartment (121, 122). Furthermore, IL‐18 showed one of the strongest associations with barrier function and AD severity (120). AD skin lesions have many skin-infiltrating cells, including CD8^+^ T cells, which can release granzyme B to cleave pro-IL-18, thus triggering inflammation activation (36). Additionally, *S. aureus*, particularly important in the pathogenesis of AD, can secrete staphylococcal enterotoxin B, which promotes IL-18 production (123). Konishi et al. reported that IL-18 played an essential role in developing AD-like dermatitis in specific transgenic mice (124). Recently, Chen JL et al. pointed out that IL-18 knockout mice reduced aggravated AD−like lesions compared to AD−like mice induced by MC903 (125). Studies have implicated that the pathogenesis of AD is mediated in part by Th2, which promotes IL-4 and IL-13 production and induces IgE (126). IL-18 enhances Th1 and Th2 responses, respectively (127); thus, IL-18 may induce IgE in AD. IL-18 increases IL-4, IL-13, and histamine levels by activating mast cells and basophils. IgE and mast cells can promote the production of a variety of inflammatory mediators (125, 128, 129). Hence, IL-18 may help evaluate the progression of AD, and IL-18 inhibition may be a prospective therapeutic target for AD.

### 4.3 Rosacea

Rosacea is a classical inflammatory dermatosis with various clinical manifestations, including erythema, telangiectasias, papules, pustules, phymatous changes in the central face, and even ocular involvement (130). Indeed, external stimuli, innate and acquired immune dysfunction combined with neurovascular dysregulation play critical roles in the pathophysiological mechanisms for rosacea (131). Among these factors, the cathelicidin LL-37 activation pathway is the best understood and most classical pathway. Under triggers such as UV radiation, demodex folliculorum, increased Toll-like receptor (TLR) 2 causes the activation and release of matrix metalloproteases (MMPs) and kallikrein 5 (KLK5), which leads to the LL-37 cleavage from its precursor. In addition, a range of pro-inflammatory conditions, including NF-κB activation, leukocyte chemotaxis, and influence on vascular endothelial growth factor (VEGF) and angiogenesis, are induced by activated LL-37 (132). Moreover, recent studies illustrated the critical roles of mast cells and Th1/Th17 cells on rosacea (133–136).

The usefulness of IL-18 in rosacea is not yet clear. Firstly, Salamon et al. found elevated serum concentration of IL-18 in rosacea patients by ELISA (137). However, Casas C et al. obtained epidermal mRNA from lesional samples by scratching the skin surface and found that compared with the control group, the rosacea group showed significantly decreased expression of IL-18 no matter the subtype (138). In 2015, Kim M et al. found that IL-18 expression in rosacea patients was upregulated by immunohistochemical staining of skin biopsies (139). They also revealed that treatment with recombinant erythroid differentiation regulator 1 (Erdr1), a negatively regulated conserved factor of IL-18, resulted in significant improvement in a rosacea-like BALB/c mouse model and downregulation of IL-18. TLR2 or TLR4 can increase IL-18 expression in different cell types; TLR2 and TLR4 can activate and then facilitate IL-18 maturation and secretion (140). IL-18 is a critical modulator of CD8^+^ T-cell activation and is a mediator of angiogenesis due to its ability to induce endothelial tube formation (141). Moreover, increased mast cells in rosacea may be associated with IL-18. Whether the IL-18 plays a role in rosacea through angiogenesis or mediating inflammation needs further study.

### 4.4 Bullous pemphigoid

Bullous Pemphigoid (BP) is one of the most common bullous autoimmune skin diseases. It is characterized by symmetric tense bullae on urticarial, erythematous, or normal skin, with histopathology of subepidermal blisters, inflammatory infiltrates, especially eosinophils in the dermis, and direct immunofluorescence showing deposition of IgG and C3 along the basement membrane region (142). BP180 is critical in the autoimmunity of bullous pemphigoid (143, 144). The dysregulation of IL-18 levels was first reported by Fang et al., who found elevated levels of IL-18 in serum, blister fluid, and lesional skin in BP patients, and a positive correlation between the serum IL-18 levels and the titers of anti-BP180-NC16A autoantibody. More importantly, they reported a dramatic decrease in mRNA expressions of the NLRP3-caspase-1-IL-18 axis components and the serum IL-18 level in BP patients after effective treatment (145). Similarly, Esmaili et al. illustrated that serum IL-18 levels were higher in untreated BP patients admitted for the first time than in control and treated BP patients (146). Besides, they also found a weak significant correlation between BP180 with IL-18 levels. Recently, Margaroli C et al. revealed that blister fluid from BP patients was enriched in multiple inflammatory factors, including IL-18 (147). Although there is no in-depth research on the relationship between IL-18 and BP, high levels of IL-18 in serum, blister fluid, and lesional skin in patients with BP indicated that IL-18 might be a key biomarker in BP.

### 4.5 Others

Lupus erythematosus is a chronic, multisystemic, genetically and environmentally induced disease with abnormal immune regulation. The patients may have cutaneous lupus erythematosus (CLE) and/or systemic lupus erythematosus (SLE) (148). CLE patients have a substantial amount of apoptotic keratinocytes in their lesions and epidermal keratinocytes, which are thought to be the target cells for immune damage (149). Wang et al. indicated that IL-18 might trigger inflammation in CLE, causing a high TNF-α response and a low IL-12 response, promoting cytokine imbalance and providing a pro-apoptotic microenvironment for keratinocytes. Thus, IL-18BP may play a vital value in the clinical therapy of CLE (149). Dermatomyositis (DM) is an autoimmune disease that affects the skin, muscles, and lungs and is characterized by a skin rash and is associated with prominent muscle weakness (150, 151). DM lesions are histologically similar to CLE lesions and are frequently confusing to distinguish. Ekholm et al. found that endothelial progenitor cells in DM, as in SLE, are characterized by phenotypic and functional abnormalities, which may be triggered by the type I IFN/IL-18 axis (152). Besides, Tsoi et al. confirmed the presence of high type I IFNs in DM skin, including IFN-κ. Their team has found that IL-18 was the only elevated cytokine in DM lesions, and that IL-18 combined with the expression of LCE2D (late cornified envelope 2D), LCE1B (late cornified envelope 1B), KRT80 (keratin 80) and TPM4 (Tropomyosin 4) to clearly differentiate DM from CLE lesions (151). Hance, IL-18 can be a valuable biomarker for DM disease activity.

Allergic contact dermatitis is also a common clinical skin condition, and since allergens are often unavoidable, improving the inflammation caused by re-exposure to allergens becomes the primary treatment. IL-18 significantly contributed to the induction of contact hypersensitivity (CHS) by increasing the recruitment of IFN-γ producing T cells to inflammation foci. Wang et al. reported that during the induction phase of CHS, LC migrating into the lymph nodes (LN) produced large amounts of functional IL-18, which acted synergistically with IL-12 to induce IFN-γ production and significantly contributed to the initiation of CHS (93). In addition, it was shown that plasma levels of free IL-18 and IL-18BP were significantly higher in eczema patients than in healthy controls and that the IL-18BP/IL-18 molar concentration ratio was reduced (153). IL-18BP not only reduced symptoms after exposure to 2,4-dinitrofluorobenzene (DNFB) but also significantly reduced inflammation in mice with previously untreated CHS (154); taken together, IL-18BP is a candidate for the therapeutic indication of allergic contact dermatitis and CHS.

## 5 Conclusion and perspective

IL-18, a member of the IL-1 family, plays a critical role in the pathogenesis of some skin inflammatory diseases. However, the specific signaling mechanism of IL-18 in inflammatory-related diseases (*e.g.* in rosacea and bullous pemphigoid) remains to be further explored. The IL-18 natural blockers, IL-18BP, has been applied successfully in an emergency case of auto-inflammatory HPS/macrophage activation syndrome. Moreover, the protective properties of recombinant IL-18BP were characterized. Overexpression of IL-18BPc in mice reduces the incidence and severity of encephalomyelitis in mice and decreases Th17 responses, and transfection of IL-18BPd-expressing plasmids *in vivo* improves murine atherosclerosis (155, 156). IL-18 opponent and IL-18BP agonist couple are promising candidates for therapy of diverse medical conditions, including acute and chronic inflammation. Accordingly, IL-18BP has shown potential therapeutic capacity in cutaneous inflammation.

## Author contributions

XYW and LW drafted the manuscript. XW and LZ edited the figures and tables. GH and XJ contributed to the critical reading and correction of the review. All authors contributed to the writing of the manuscript and approved the submitted version.
